# Echogenic foci in thyroid nodules: diagnostic performance with combination of TIRADS and echogenic foci

**DOI:** 10.1186/s12880-019-0328-2

**Published:** 2019-04-04

**Authors:** Su Min Ha, Yun Jae Chung, Hye Shin Ahn, Jung Hwan Baek, Sung Bin Park

**Affiliations:** 1 Department of Radiology, Chung-Ang University Hospital, Chung-Ang University College of Medicine, 102, Heukseok-ro, Dongjak-gu, Seoul, 06973 South Korea; 2 Department of Internal Medicine, Chung-Ang University Hospital, Chung-Ang University College of Medicine, 102, Heukseok-ro, Dongjak-gu, Seoul, 06973 Republic of Korea; 3 Department of Radiology and Research Institute of Radiology, University of Ulsan College of Medicine, Asan Medical Center, 86 Asanbyeongwon-Gil, Songpa-gu, Seoul, 05505 South Korea; 4 Department of Radiology, Seoul National College of Medicine, 101 Daehak-ro, Jongno-gu, Seoul, 110-744 Republic of Korea

**Keywords:** Thyroid nodules, Ultrasound, Thyroid cancer, Thyroid TIRADS, Echogenic foci

## Abstract

**Background:**

The malignancy risks of various echogenic foci in thyroid nodules are not consistent. The association between malignancy and echogenic foci and various Thyroid Imaging Reporting and Data System (TIRADS) in thyroid nodules has not been evaluated. We evaluated the malignancy probability and diagnostic performance of thyroid nodules with various echogenic foci and in combination with TIRADS.

**Methods:**

This retrospective study was approved by Institutional Review Board. The data were retrospectively collected from January 2013 to December 2014. In total, 954 patients (mean age, 50.8 years; range, 13–86 years) with 1112 nodules were included. Using χ^2^ test, we determined the prevalence of benign and malignant nodules among those with and without echogenic foci; we associated each of 6 echogenic foci types with benign and malignant nodules. Diagnostic performance was compared between the 6 types alone and in combination with various TIRADS.

**Results:**

Among 1112 nodules, 390 nodules (35.1%) were found to have echogenic foci, and 722 nodules (64.9%) were not. Among nodules with echogenic foci, 254 nodules (65.1%) were malignant. The punctate echogenic foci with comet-tail artifact showed malignancy rate of 77.8% in solid and predominantly solid nodules. Our study demonstrated relatively low PPV (33.3–56.4%) in nodules with large echogenic foci without shadowing, macrocalcification, and peripheral curvilinear or eggshell echogenic foci with or without shadowing. However, when combined with high suspicion category of TIRADS, PPV increased to 50.0–90.9%.

**Conclusion:**

Combination with TIRADS with different types of echogenic foci offer better stratification of the malignancy risk.

**Electronic supplementary material:**

The online version of this article (10.1186/s12880-019-0328-2) contains supplementary material, which is available to authorized users.

## Background

Although widespread use of ultrasonography (US) has exponentially increased thyroid nodule detection to about 19.0–67.0%, malignancy is found in only about 9.0–15.0% of nodules evaluated using fine-needle aspiration (FNA) [1–3]. To minimize potential harm from overuse of FNA, the Thyroid Imaging Reporting and Data System (TIRADS) was developed for thyroid nodule risk stratification [4–6]. One such model was recently published by the Korean Society of Thyroid Radiology (KSThR) named the Korean TIRADS [7], and was validated prospectively in a multi-center study [8]. The American Thyroid Association (ATA) management guidelines for thyroid nodules also stratified the risk of malignancy into five categories [5]. Meanwhile, Choi et al. [9] developed a web-based automatic scoring risk stratification system using US characteristics. This system also classifies nodules according to various guidelines, such as the French TIRADS [10], ATA guidelines [5], and Korean TIRADS [7]. The various TIRADS include “echogenic foci” as a criterion for differentiating benign and malignant thyroid nodules. Echogenic foci within the thyroid nodule are common on US with 14.0–55.0% incidence [11–14] and 29.0–59.0% malignancy rate [11, 13–16]. Indeed, there are various patterns of echogenic foci [12, 15, 17], but are often poorly defined and simply referred to as calcifications. The clinical significance of other types of echogenic foci is not yet clear, except for punctate echogenic foci what many authors call as microcalcifications [18–21]. Microcalcifications are generally accepted as the most reliable indicator of malignancy because they mostly represent psammoma bodies [12].

The recently published American College of Radiology (ACR) TIRADS defined echogenic foci into five categories: punctate foci with no posterior artifact, echogenic foci with small comet-tail artifact (≤ 1 mm long), echogenic foci with large comet-tail artifact (> 1 mm long), peripheral echogenic foci with acoustic shadowing, and clumplike echogenic foci with acoustic shadowing [20]. They insisted that echogenic foci with small comet-tail artifact in solid hypoechoic nodule should be distinguished from the large comet-tail artifacts in the cystic components of a thyroid nodule [22]. And large comet-tail artifacts in hypoechoic nodules to be viewed with suspicion. According to Korean-TIRADS developed by KSThR, the malignancy risk of microcalcification is high in the solid hypoechoic nodules, but intermediate risk in the partially cystic and iso- and hyperechoic nodules .

The malignancy risks of various echogenic foci in thyroid nodules are not consistent; evidence is limited with variable value. Furthermore, the association between malignancy and echogenic foci and various TIRADS in thyroid nodules has not been evaluated. Our study evaluated the malignancy probability and diagnostic performance in differentiating benign and malignant nodules with various types of echogenic foci and when combined with various TIRADS.

## Methods

This retrospective study was approved by the Institutional Review Board; informed consent was waived for data evaluation. Written informed consent for routine thyroid US and US-guided procedures was obtained from all patients before each US examination.

### Study population

The patient cohort was retrospectively collected in patients undergoing US from January 2013 to December 2014. A total of 2703 consecutive nodules (≥ 5 mm) were selected for a database of thyroid nodules that underwent US-guided core-needle biopsy (CNB) or FNA. Among them, 1591 nodules with indeterminate or non-diagnostic results and without follow-up for final diagnosis were excluded. Finally, a total of 954 patients (mean age, 50.8 years; range, 13–86 years) with 1112 nodules (≥ 5 mm) were included in our study. For each thyroid nodule, the final diagnosis was determined either by histopathology or radiological follow-up. For malignant nodules (*n* = 417), the pathological diagnosis was confirmed by surgery (*n* = 413) or CNB (*n* = 4) [23]. For benign nodules (*n* = 695), the pathological diagnosis was confirmed by surgery (*n* = 65), repeated FNA or CNB at least twice with benign results (*n* = 41), or a benign result on FNA or CNB with no change or reduced size seen on follow-up US more than > 12 months later (*n* = 589). The interpretation of FNA was based on the Bethesda system for reporting thyroid cytopathology [24], and the 6 categories of a CNB pathology reporting system were used for interpretation of CNB [25].

### Ultrasound examination

Ultrasound images for the evaluation of thyroid nodules were obtained using an iU22 ultrasound system (Philips Healthcare, Bothell, WA) equipped with a 50-mm linear array transducer with a bandwidth of 7–12 MHz. The scanning protocol in all cases included both transverse and longitudinal real-time imaging of thyroid nodules. US images were retrospectively reviewed by two radiologists (S.M.H., H.S.A) who had 8–10 years of experience in performing thyroid US. Images were reviewed with no previous knowledge of the biopsy result or final diagnosis and assessed the US features of thyroid nodules-internal content, echogenicity, margin, shape, presence or absence echogenic foci and comet-tail artifact. We excluded vascularity as a criterion.

The internal content of a nodule was categorized as solid (pure solid or nearly entirely solid), predominantly solid (< 50% of the cystic portion), predominantly cystic (> 50% of the cystic portion), or cystic (no obvious solid content). Echogenicity of the solid portion was classified as hyper- or iso-echogenicity, hypoechogenicity, or marked hypoechogenicity. When the echogenicity of the nodule was similar to that of the surrounding thyroid parenchyma, it was classified as isoechogenicity. Hypoechogenicity was defined as decreased echogenicity compared to the thyroid parenchyma. Marked hypoechogenicity was defined as decreased echogenicity compared to that of the strap muscles. The nodule shape was categorized as follows: ovoid to round (when the anteroposterior diameter of the nodule was equal to or less than its transverse diameter on a transverse or longitudinal plane); taller than wide (when the anteroposterior diameter of a nodule was longer than its transverse diameter on a transverse or longitudinal plane); or irregular (when a nodule was neither ovoid to round nor taller than wide). Margins were classified as well-defined smooth, microlobulated or spiculated, or ill-defined.

Nodules that did not have echogenic foci were kept for overall comparison. The nodules with echogenic foci were classified into the following 6 types: Type 1.Punctate echogenic foci (≤ 1 mm) with or without posterior shadowing, Type 2. Punctate echogenic foci with comet-tail artifact, Type 3. Large echogenic foci (> 1 mm) without shadowing, Type 4. Macrocalcification (defined as large echogenic foci (> 1 mm) with shadowing), Type 5. Peripheral curvilinear or eggshell echogenic foci with or without shadowing, and Type 6. More than one type of echogenic foci. We calculated the probability of malignancy using various malignant risk systems, such as web-based TIRADS, K-TIRADS, ATA guidelines [5], and TIRADS of Russ et al. [26]. Suspicious nodules were defined as those with a score of ≥8 with the web-based TIRADS, high suspicion with K-TIRADS, high suspicion with the ATA guidelines [5], and a score of ≥4B with the TIRADS proposed by Russ et al. [26].

### Data and statistical analysis

Multivariate logistic regression analysis was used to estimate the malignancy risk associated with US findings in thyroid nodules with and without echogenic foci. Using the χ^2^ test, we determined the overall prevalence of benign and malignant nodules among those with 6 echogenic foci types and by various guidelines. Diagnostic performance was evaluated by echogenic foci types alone, and in combination with various TIRADS. We calculated the sensitivity, specificity, negative predictive value (NPV), positive predictive value (PPV) and accuracy. The PPV between two classifications (1. nodules with echogenic foci and high suspicion TIRADS category, 2. nodules with echogenic foci and non-high suspicion TIRADS category) was compared using χ^2^ or Fisher’s exact test. All statistical analyses were carried out using SPSS version 23.0 (IBM Corp., Armonk, NY, USA). Two-tailed *p* values < 0.05 were considered to be statistically significant.

## Result

Among the 1112 nodules, 390 (35.1% [390 of 1112]) were found to have echogenic foci on US, and 722 (64.9% [722 of 1112]) were not. Among 390 nodules with echogenic foci, 254 (65.1% [254 of 390]) were malignant, and 136 (34.9% [136 of 390]) were benign. Among 722 nodules without echogenic foci, 163 (22.6% [163 of 722]) were malignant, and 559 (77.4% [559 of 722]) were benign. There was a significant statistical difference (22.6% vs 65.1%, *p* < 0.001) in the prevalence of malignant lesions between nodules with and those without echogenic foci. In nodules with echogenic foci, the taller than wide shape (*p* = 0.026), spiculated margin (*p* < 0.001), marked hypoechogenicity (*p* < 0.001) were US features that showed significant difference between benign and malignant nodules. Solid (*n* = 352, 90.3%) or predominantly solid (*n* = 38, 9.7%) compositions were the most common features in nodules with echogenic foci (Additional file 1: Table S1). In nodules without echogenic foci, spiculated margin (*p* < 0.001) and marked hypoechogenicity (*p* < 0.001) were US features that showed significant difference. Among malignant lesions with echogenic foci (*n* = 254), 205 (80.7% [205 of 254]) were papillary carcinoma, 6 (2.4% [6 of 254]) were follicular carcinoma, 32 (12.6% [32 of 254]) were follicular variant papillary carcinoma, and 11 (4.3% [11 of 254]) were other histologic types such as medullary carcinoma (Additional file 1: Table S2). Benign lesions varied widely and included follicular adenoma, lymphocytic thyroiditis, and nodular hyperplasia.

**Fig. 1:**
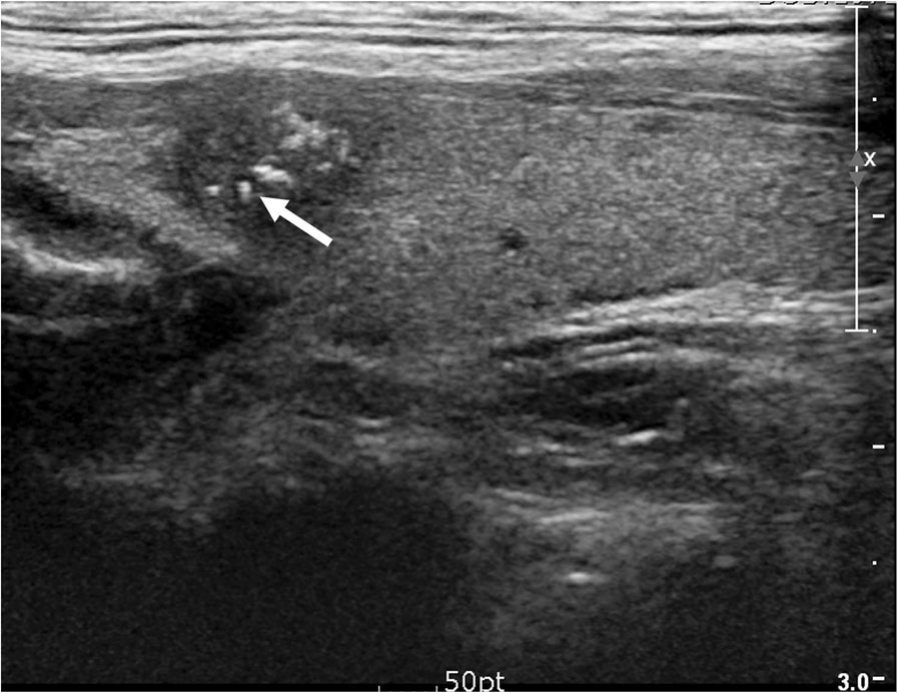
Echogenic foci associated with malignancy. 41-year-old male with thyroid nodule. Ultrasound image shows a 1.0 cm hypoechoic, solid nodule with spiculated margin. Multiple echogenic foci (Type 6) are present, including echogenic foci with comet-tail artifact (Type 2, arrow). Biopsy result was papillary carcinoma and was confirmed at surgery

The prevalence of benign versus malignant nodules for each 6 types of echogenic foci is summarized in Table 1. All were associated with a relatively high prevalence of malignancy (33.3–77.8%). Punctate echogenic foci with or without posterior shadowing was the most commonly encountered (53.1% [207 of 390]) (Fig. 1). Peripheral curvilinear or eggshell echogenic foci with or without shadowing was the least seen (5.4% [21 of 390]). Table 2 demonstrates the malignancy probability of thyroid nodules with suspicious US features according to various malignant risk stratification systems. Among the various TIRADS, the malignancy probability ranged from 70.1–96.2%, wherein the ATA guideline exhibited the highest sensitivity.

**Table 1:** Rate of Malignancy by Thyroid Nodule Echogenic Foci Type

	Total (*n* = 390)	Benign (*n* = 136)	Malignant (*n* = 254)	*P* value
Type 1	207 (53.1)	54(26.1)	153 (73.9)	< 0.001
Type 2	54 (13.8)	12(22.2)	42(77.8)	0.452
Type 3	94 (24.1)	41 (43.6)	53 (56.4)	0.041
Type 4	27 (6.9)	16 (59.3)	11 (40.7)	0.006
Type 5	21(5.4)	14 (66.7)	7 (33.3)	0.002
Type 6	41 (10.5)	11 (26.8)	30 (73.2)	0.253

Data indicate the number of lesions

Numbers in parentheses indicate percentages

Type 1 = Punctate echogenic foci (≤ 1 mm) with or without posterior shadowing

Type 2 = Punctate echogenic foci with comet-tail artifact

Type 3 = Large echogenic foci (> 1 mm) without shadowing

Type 4 = Macrocalcification (defined as large echogenic foci (> 1 mm) with shadowing)

Type 5 = Peripheral curvilinear or eggshell echogenic foci with or without shadowing

Type 6 = Nodules with more than one type of echogenic foci

**Table 2:** Comparison of Malignancy Rate According to Various Guidelines

	TIRADS	Suspicious nodule on US	Confirmed as cancer	Malignancy rate
Total (*n* = 390)	Web-based TIRADS(≥ 8)	199 (51.0)	178 (70.1)	70.1%
	K-TIRADS High Suspicion	226 (57.9)	198 (78.0)	78.0%
	ATA guidelines High Suspicion	235 (60.3)	205 (96.2)	96.2%
	Russ et al. (≥ 4B)	278 (71.3)	217 (85.4)	85.4%

Data indicate the number of lesions

Numbers in parentheses indicate percentages

*TIRADS* thyroid image reporting and data system, *ATA* american thyroid association

**Fig. 2:**
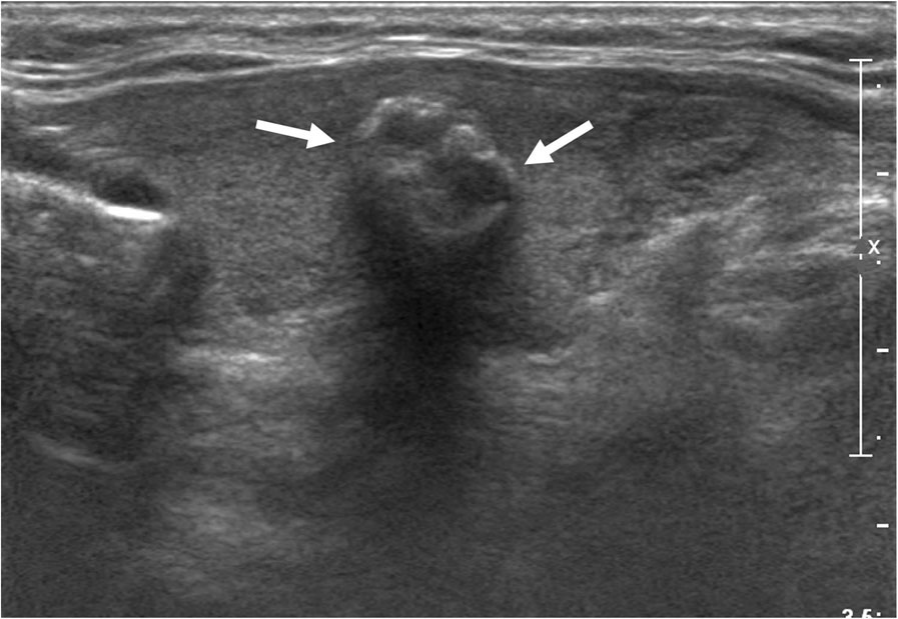
38-year-old woman with 1.3 cm solid hypoechoic thyroid nodule. Ultrasound image shows macrocalfication with posterior shadowing (Type 4, arrows). Biopsy finding was papillary carcinoma and confirmed at surgery

**Fig. 3:**
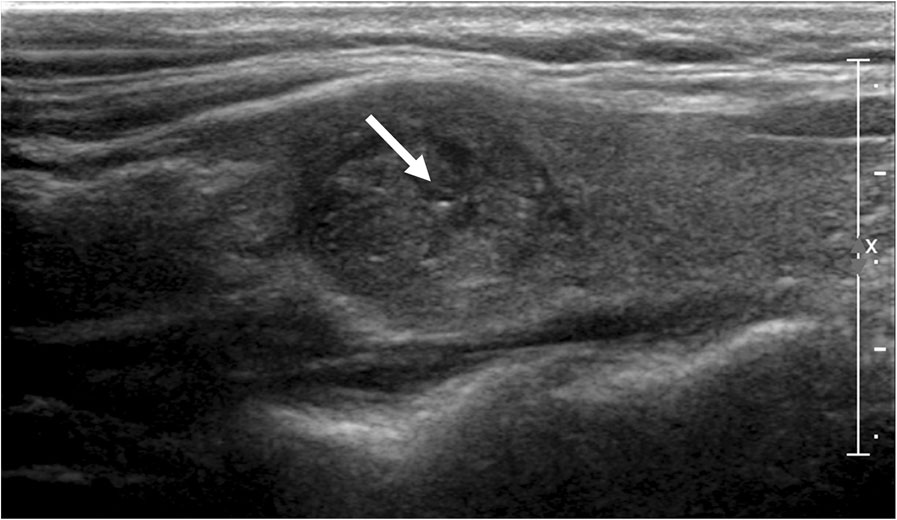
Echogenic foci associated with malignancy. 36-year-old female with thyroid nodule. Ultrasound image shows a 1.8 cm hypoechoic, solid nodule. Echogenic foci with no posterior acoustic artifact (Type 1, arrow). Biopsy result was papillary carcinoma and was confirmed at surgery

**Fig. 4:**
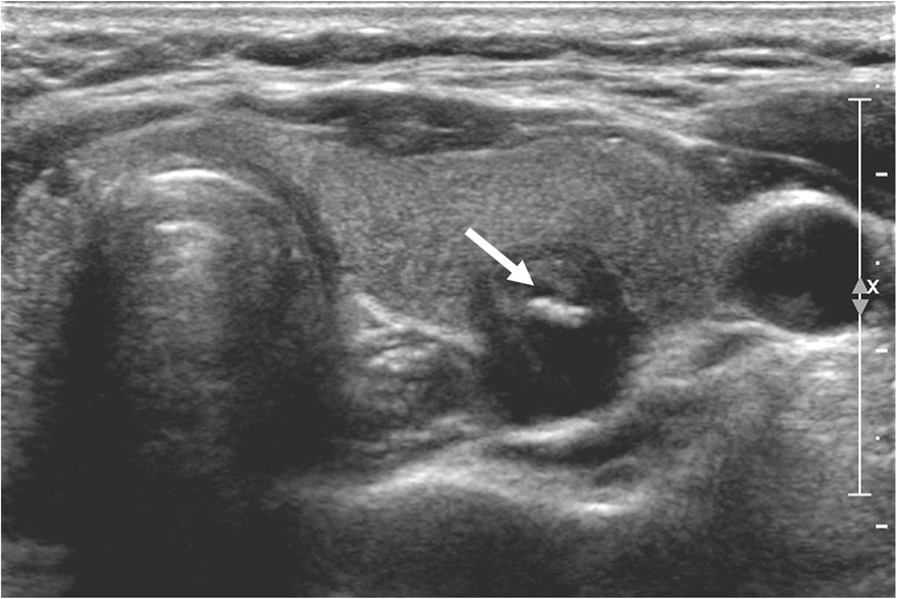
Echogenic foci associated with malignancy. 51-year-old female with thyroid nodule. Ultrasound image shows a 1.1 cm hypoechoic, solid nodule with large echogenic foci (> 1 mm) without shadowing (Type 3, arrow). Biopsy result was papillary carcinoma and was confirmed at surgery

**Fig. 5:**
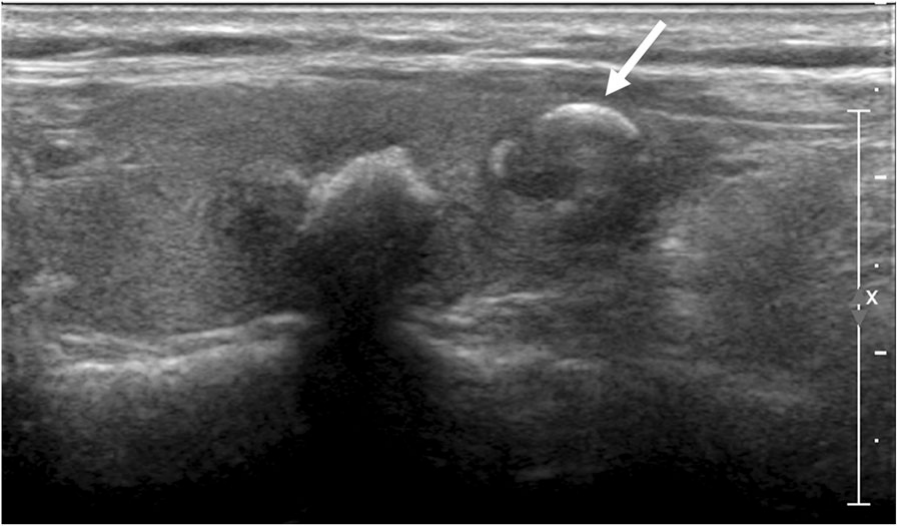
Echogenic foci associated with malignancy. 70-year-old female with thyroid nodule. Ultrasound image shows a 1.0 cm hypoechoic, solid nodule with peripheral curvilinear echogenic foci (Type 5, arrow). Biopsy result was papillary carcinoma and was confirmed at surgery

Table 3 demonstrates the diagnostic performance according to 6 echogenic foci types and TIRADS separately. In regard of echogenic foci type alone, punctate echogenic foci with or without posterior shadowing, punctate echogenic foci with comet-tail artifact, and more than one type of echogenic foci showed the high PPV (73.9, 77.8, and 73.2% respectively). Macrocalcification and peripheral curvilinear or eggshell echogenic foci showed relatively low PPV, 40.7 and 33.3% respectively. However, when combined with high suspicion categories of various TIRADS (Table 4), PPV improved in all, especially the improvement was notable in macrocalcification and peripheral curvilinear or eggshell echogenic foci types, to a range of 50.0–80.0% (Fig. 2). Large echogenic foci without shadowing alone showed PPV of 56.4%, and when combined with the web-based scoring system, it also improved its PPV to 90.9%. Meanwhile, when we combined 6 types of echogenic foci with non-high suspicion TIRAD category, the PPV decreased to a range of 10.5–57.1% (Table 5). In comparison of PPV between nodules with echogenic foci and high suspicion or non-high suspicion TIRADS category, all showed statistically significant difference except for punctate echogenic foci with comet-tail artifact combined with K-TIRADS (85.0% vs 57.1%, *p* = 0.057) and ATA guideline (84.8% vs 33.3% *p* = 0.083), macrocalcification combined with the web-based scoring system (50.0% vs 40.0%, *p* > 0.999), K-TIRADS (75.0% vs 34.8%, *p* = 0.273), ATA guideline (75.0% vs 16.7%, *p* = 0.191), Russ et al. (66.7% vs 33.3%, *p* = 0.187), and peripheral curvilinear or eggshell echogenic foci combined with web-based scoring system (66.7% vs 20.0%, *p* = 0.120), ATA guideline (80.0% vs 16.7%, *p* = 0.080) and Russ et al. (66.7% vs 20.0%, *p* = 0.120) (Table 6) (Figs. 3, 4 and 5).

**Table 3:** Diagnostic Performance of US Characteristics for the Prediction of Malignancy in Thyroid Nodules with Echogenic Foci alone and TIRADS alone

		Total	Benign	Malignant	Diagnostic Performance				
					Sensitivity	Specificity	Accuracy	PPV	NPV
Echogenic Foci	Type 1	207	54	153	60.2	60.3	60.3	73.9	44.8
	Type 2	54	12	42	27.5	77.8	40.6	77.8	27.5
	Type 3	94	41	53	20.9	69.9	37.9	56.4	32.1
	Type 4	27	16	11	4.3	88.2	33.6	40.7	33.1
	Type 5	21	14	7	2.8	89.7	33.1	33.3	33.1
	Type 6	41	11	30	11.8	91.9	39.7	73.2	35.8
TIRADS	Web-based TIRADS(≥ 8)	199	21	178	70.1	84.6	75.1	89.4	60.2
	K-TIRADS High Suspicion	226	28	198	78.0	79.4	78.5	87.6	65.9
	ATA guidelines High Suspicion	235	30	205	96.2	56.5	86.5	87.2	83.0
	Russ et al. (≥ 4B)	278	61	217	85.4	55.1	74.9	78.1	67.0

*TIRADS* thyroid image reporting and data system, *ATA* american thyroid association, *PPV* positive predictive value, *NPV* negative predictive value

Type 1 = Punctate echogenic foci (≤ 1 mm) with or without posterior shadowing

Type 2 = Punctate echogenic foci with comet-tail artifact

Type 3 = Large echogenic foci (> 1 mm) without shadowing

Type 4 = Macrocalcification (defined as large echogenic foci (> 1 mm) with shadowing)

Type 5 = Peripheral curvilinear or eggshell echogenic foci with or without shadowing

Type 6 = Nodules with more than one type of echogenic foci

**Table 4:** Diagnostic Performance of US Characteristics for the Prediction of Malignancy in Thyroid Nodules with Echogenic foci Types with TIRADS Combination (High suspicion)

Echogenic foci	TIRADS	Total	Benign	Malignant	Diagnostic Performance				
					Sensitivity	Specificity	Accuracy	PPV	NPV
Type 1	+ Web-based TIRADS (≥ 8)	148	14	134	52.8	89.7	65.6	90.5	50.4
	+ K-TIRADS High Suspicion	156	19	137	53.9	86.0	65.1	87.8	50.0
	+ ATA High Suspicion	163	21	142	66.7	69.6	67.4	87.1	40.3
	+ Russ et al. (≥ 4B)	189	41	148	58.3	69.9	62.3	78.3	47.3
Type 2	+ Web-based TIRADS (≥ 8)	37	3	34	22.2	94.4	41.1	91.9	30.0
	+K-TIRADS High Suspicion	40	6	34	22.2	88.9	39.6	85.0	28.7
	+ ATA High Suspicion	46	7	39	27.1	75.9	35.3	84.8	17.3
	+ Russ et al. (≥ 4B)	47	8	39	25.5	85.2	41.1	83.0	28.8
Type 3	+ Web-based TIRADS (≥ 8)	22	2	20	7.9	98.5	39.5	90.9	36.4
	+K-TIRADS High Suspicion	37	6	31	12.2	95.6	41.3	83.8	36.8
	+ ATA High Suspicion	38	6	32	15.0	91.3	33.7	84.2	25.8
	+ Russ et al. (≥ 4B)	49	13	36	14.2	90.4	40.8	73.5	36.1
Type 4	+ Web-based TIRADS (≥ 8)	2	1	1	0.4	99.3	34.9	50.0	34.8
	+K-TIRADS High Suspicion	4	1	3	1.2	99.3	35.4	75.0	35.0
	+ ATA High Suspicion	4	1	3	1.4	98.6	25.2	75.0	24.5
	+ Russ et al. (≥ 4B)	6	2	4	1.6	98.5	35.4	66.7	34.9
Type 5	+ Web-based TIRADS (≥ 8)	6	2	4	1.6	98.5	35.4	66.7	34.9
	+K-TIRADS High Suspicion	5	1	4	1.6	99.3	35.6	80.0	35.1
	+ ATA High Suspicion	5	1	4	1.9	98.6	25.5	80.0	24.5
	+ Russ et al. (≥ 4B)	6	2	4	1.6	98.5	35.4	66.7	34.9
Type 6	+ Web-based TIRADS (≥ 8)	21	2	19	7.5	98.5	39.2	90.5	36.3
	+K-TIRADS High Suspicion	24	1	23	9.1	99.3	40.5	95.8	36.9
	+ ATA High Suspicion	25	1	24	11.3	98.6	32.6	96.0	26.5
	+ Russ et al. (≥ 4B)	28	3	25	9.8	97.8	40.5	89.3	36.7

*TIRADS* thyroid image reporting and data system, *ATA* american thyroid association, *PPV* positive predictive value, *NPV* negative predictive value

Type 1 = Punctate echogenic foci (≤ 1 mm) with or without posterior shadowing

Type 2 = Punctate echogenic foci with comet-tail artifact

Type 3 = Large echogenic foci (> 1 mm) without shadowing

Type 4 = Macrocalcification (defined as large echogenic foci (> 1 mm) with shadowing)

Type 5 = Peripheral curvilinear or eggshell echogenic foci with or without shadowing

Type 6 = Nodules with more than one type of echogenic foci

**Table 5:** Diagnostic Performance of US Characteristics for the Prediction of Malignancy in Thyroid Nodules with Echogenic foci Types with TIRADS Combination (Low suspicion)

Echogenic foci	TIRADS	Total	Benign	Malignant	Diagnostic Performance				
					Sensitivity	Specificity	Accuracy	PPV	NPV
Type 1	+ Web-based TIRADS (< 8)	59	40	19	7.5	70.6	29.5	32.2	29.0
	+ K-TIRADS^a^	51	35	16	6.3	74.3	30.0	31.4	29.8
	+ ATA^a^	10	8	2	0.9	88.4	22.3	20.0	22.4
	+ Russ et al. (< 4B)	18	13	5	2.0	90.4	32.8	27.8	33.1
Type 2	+ Web-based TIRADS (< 8)	17	9	8	5.2	83.3	27.1	47.1	23.7
	+ K-TIRADS^a^	14	6	8	5.2	88.9	25.6	57.1	24.9
	+ ATA^a^	3	2	1	0.7	93.1	16.2	33.3	15.9
	+ Russ et al. (<4B)	7	4	3	2.0	92.6	25.6	42.9	25.0
Type 3	+ Web-based TIRADS (< 8)	72	39	33	13.0	71.3	33.3	45.8	30.5
	+ K-TIRADS^a^	57	35	22	8.7	74.3	31.5	38.6	30.3
	+ ATA^a^	19	17	2	0.9	75.4	19.1	10.5	19.8
	+ Russ et al. (<4B)	45	28	17	6.7	79.4	32.1	37.8	31.3
Type 4	+ Web-based TIRADS (< 8)	25	15	10	3.9	89.0	33.6	40.0	33.2
	+ K-TIRADS^a^	23	15	8	3.1	89.0	33.1	34.8	33.0
	+ ATA^a^	6	5	1	0.5	92.8	23.0	16.7	23.2
	+ Russ et al. (<4B)	21	14	7	2.8	89.7	33.1	33.3	33.1
Type 5	+ Web-based TIRADS (< 8)	15	12	3	1.2	91.2	32.6	20.0	33.1
	+ K-TIRADS^a^	16	13	3	1.2	90.4	32.3	18.8	32.9
	+ ATA^a^	6	5	1	0.5	92.8	23.0	16.7	23.2
	+ Russ et al. (<4B)	15	12	3	1.2	91.2	32.6	20.0	33.1
Type 6	+ Web-based TIRADS (< 8)	20	9	11	4.3	93.4	35.4	55.0	34.3
	+ K-TIRADS^a^	17	10	7	2.8	92.6	34.1	41.2	33.8
	+ ATA^a^	6	4	2	0.9	94.2	23.8	33.3	23.6
	+ Russ et al. (< 4B)	13	8	5	2.0	94.1	34.1	38.5	34.0

*TIRADS* thyroid image reporting and data system, *ATA* american thyroid association, *PPV* positive predictive value, *NPV* negative predictive value

^a^Other than High suspicion category

Type 1 = Punctate echogenic foci (≤ 1 mm) with or without posterior shadowing

Type 2 = Punctate echogenic foci with comet-tail artifact

Type 3 = Large echogenic foci (> 1 mm) without shadowing

Type 4 = Macrocalcification (defined as large echogenic foci (> 1 mm) with shadowing

Type 5 = Peripheral curvilinear or eggshell echogenic foci with or without shadowing

Type 6 = Nodules with more than one type of echogenic foci

**Table 6:** Comparison of Positive Predictive Value in Thyroid Nodules with Echogenic foci Types with different TIRADS Category Combination

Echogenic foci	TIRADS	PPV	TIRADS	PPV	*P* value
Type 1	Web-based TIRADS (≥ 8)	90.5	Web-based TIRADS (< 8)	32.2	< 0.001
	K-TIRADS High Suspicion	87.8	K-TIRADS^a^	31.4	< 0.001
	ATA High Suspicion	87.1	ATA^a^	20.0	< 0.001
	Russ et al. (≥ 4B)	78.3	Russ et al. (< 4B)	27.8	< 0.001
Type 2	Web-based TIRADS (≥ 8)	91.9	Web-based TIRADS (< 8)	47.1	< 0.001
	K-TIRADS High Suspicion	85.0	K-TIRADS^a^	57.1	0.579
	ATA High Suspicion	84.8	ATA^a^	33.3	0.083
	Russ et al. (≥ 4B)	83.0	Russ et al. (<4B)	42.9	0.036
Type 3	Web-based TIRADS (≥ 8)	90.9	Web-based TIRADS (< 8)	45.8	< 0.001
	K-TIRADS High Suspicion	83.8	K-TIRADS^a^	38.6	< 0.001
	ATA High Suspicion	84.2	ATA^a^	10.5	< 0.001
	Russ et al. (≥ 4B)	73.5	Russ et al. (<4B)	37.8	< 0.001
Type 4	Web-based TIRADS (≥ 8)	50.0	Web-based TIRADS (< 8)	40.0	> 0.999
	K-TIRADS High Suspicion	75.0	K-TIRADS^a^	34.8	0.237
	ATA High Suspicion	75.0	ATA^a^	16.7	0.191
	Russ et al. (≥ 4B)	66.7	Russ et al. (<4B)	33.3	0.187
Type 5	Web-based TIRADS (≥ 8)	66.7	Web-based TIRADS (< 8)	20.0	0.120
	K-TIRADS High Suspicion	80.0	K-TIRADS^a^	18.8	0.025
	ATA High Suspicion	80.0	ATA^a^	16.7	0.080
	Russ et al. (≥ 4B)	66.7	Russ et al. (<4B)	20.0	0.120
Type 6	Web-based TIRADS (≥ 8)	90.5	Web-based TIRADS (< 8)	55.0	0.015
	K-TIRADS High Suspicion	95.8	K-TIRADS^a^	41.2	< 0.001
	ATA High Suspicion	96.0	ATA^a^	33.3	< 0.001
	Russ et al. (≥ 4B)	89.3	Russ et al. (< 4B)	38.5	< 0.001

*TIRADS* thyroid image reporting and data system, *ATA* american thyroid association, *PPV* positive predictive value

^a^Other than High suspicion category

*TIRADS* Thyroid image reporting and data system, *ATA* American Thyroid Association, *PPV* Positive Predictive Value

Type 1 = Punctate echogenic foci (≤ 1 mm) with or without posterior shadowing

Type 2 = Punctate echogenic foci with comet-tail artifact

Type 3 = Large echogenic foci (> 1 mm) without shadowing

Type 4 = Macrocalcification (defined as large echogenic foci (> 1 mm) with shadowing

Type 5 = Peripheral curvilinear or eggshell echogenic foci with or without shadowing

Type 6 = Nodules with more than one type of echogenic foci

## Discussion

Among the 6 types of echogenic foci we defined, the most common type was punctate echogenic foci with or without posterior acoustic shadowing, referred to as microcalcifications by many authors. This was associated with a high malignancy rate (73.9%). Interestingly, punctate echogenic foci with comet-tail artifact were associated with high prevalence of malignancy (77.8%). Our result indicates that punctate echogenic foci with comet-tail artifact in solid or predominantly solid nodules should be viewed with suspicion, in accordance with the previous study [22]. Moreover, our study demonstrated 3 types of echogenic foci (large echogenic foci without shadowing, macrocalcification, and peripheral curvilinear or eggshell echogenic foci) with relatively low PPV, 33.3–56.4%. However, when we combined these echogenic foci with various high suspicion categories of TIRADS, the PPV increased. In contrast, when nodules with echogenic foci were combined with non-high suspicion TIRAD category, the PPV decreased. Various TIRADS may further stratify the malignancy risk of nodules with each echogenic foci types. Thus, the combined assessment of TIRADS and echogenic foci is more predictive of malignancy than echogenic foci alone.

Several TIRADS have been developed for malignancy risk stratification [4, 6, 27] that incorporate US features to categorize thyroid nodules and recommend cytological diagnosis. Korean TIRADS [7] and the ATA guidelines for thyroid nodules stratified the risk of malignancy into several categories based on US patterns [5]. Meanwhile, several attempts have been made to convert this “pattern-based” approach to a “score-based” approach. Representatively, Choi et al. [9] developed a web-based scoring risk stratification system using US characteristics. The TIRADSalso has been recently combined with cytology or scoring systems in management of indeterminate nodules [28, 29]. Therefore, we hypothesized combining TIRADS with variable echogenic foci would demonstrate promising capacity to distinguish thyroid malignancy with clinical importance. Indeed, the relationship between risk of malignancy and echogenic foci has been debatable; there have been several attempts to combine several US features with indeterminate echogenic foci of thyroid nodule in predicting the malignancy probability [30, 31]. Frates et al. [30] reported that coarse or rim calcifications double the risk of malignancy as compared with similar nodules without and its risk increases when a nodule is solitary and solid. Kim et al. [31] showed that 82.8% of malignancy with macrocalcifications are based on triple criteria (marked hypoechogenicity, irregular or microlobulated margin, and a taller than wide shape); the risk of malignancy was 82.8% in cases with at least 1 of the triple criteria, whereas 34.5% in cases with no suspicious sonographic finding. Similarly, in our study, by combining various types of echogenic foci with non-high suspicion and high suspicion categories of TIRADS, we observed the changes in diagnostic performance of differentiating benign and malignant thyroid nodules. Combination of TIRADS is more useful for prediction of malignancy risk compared to individual US feature of echogenic foci.

Previous studies have indicated that echogenic foci are more common in malignant thyroid nodules (incidence of 26.0–54.0%) than in benign lesions (8.0–32.0%) [15, 16, 32]. This is reflected in our study which revealed significant difference in the prevalence of malignant lesions with and without echogenic foci (nodules with echogenic foci, 65.1% [254 of 390]; nodules without echogenic foci, 22.6% [163 of 722]; *p* < 0.001). Although the presence of echogenic foci is highly suggestive of malignancy [12, 17, 18, 20, 21], the clinical significance of specific patterns of echogenic foci is unclear. Echogenic foci range from classic microcalcification associated with papillary thyroid carcinoma to the echogenic foci with comet-tail artifact in benign colloid nodules. Beland et al. [33] found it difficult to categorize 62.0% of the nodules into either of these echogenic foci; they also found that non-shadowing brightly echogenic linear foci with or without comet-tail artifact is associated with benign nodules. Additionally, the posterior comet-tail artifacts of echogenic foci in cystic nodules have been strongly associated with benignity. Ahuja et al. [34] observed 100 complex cystic nodules with echogenic foci and associated comet-tail artifact were 100% sensitive and specific for benignity. However, some describe comet-tail artifacts in malignant lesions [33, 35]. Malhi et al. [22] emphasized that comet-tail artifact in hypoechoic nodules should be viewed with suspicion and distinguished from artifacts in cystic components. Likewise, the punctate echogenic foci with comet-tail artifact in solid and predominantly solid nodules showed malignancy rate of 77.8% in our study. The echogenic foci with comet-tail artifacts in the solid portion are not specific for a benign nodule. Indeed, the malignancy risk of punctate echogenic foci of nodule depend on solidity and composition, and comet-tail artifact show relatively high malignancy risk when it is located within hypoechoic solid component. If the proportion of isoechoic or partially cystic nodules with punctate echogenic foci is high or nodules showing echogenic foci within the cystic content is high, the malignancy rate will decrease. Although the exact location of the echogenic foci was not analyzed in this study, the higher predictive value for malignancy by combined TIRADS evaluation may explain the different malignancy risk according to the other US characteristics of nodule. In real practice, we do not recommend for biopsy on the presence of echogenic foci of thyroid nodule alone; rather, we analyze the nodule characteristics such as composition, margin, echogenicity, and size for nodule sampling. We agree that biopsy should be determined after thorough interpretation of thyroid nodule. Nonetheless, our study assessed the association of thyroid malignancy with various echogenic foci and TIRADS in diagnosing thyroid nodule; any type of detected echogenic foci deserves further investigation with suspicion for possible malignancy and consideration of accompanying US features. In addition, although FNA is a good diagnostic tool with a sensitivity of 71.0–93.0% and a specificity of 96.0% [11, 36], the diagnostic material from calcified nodules is difficult to assess with inadequate results of about 26.8% [37]. Our study may provide useful information about thyroid nodules with echogenic foci in cases with inadequate cytological results.

The nodules with more than one type of echogenic foci were proven to increase the risk of malignancy from 11.2% for those with a single type to 24.7% for those having more than one type [22]. Indeed, we encounter many nodules with more than one type of echogenic foci. Several investigations suggest detection of macrocalcifications as well as microcalcifications should increase suspicion of thyroid carcinoma [11, 13, 14, 30, 38]. Similarly, in our study, nodules with more than one type of echogenic foci showed a malignancy rate of 73.2%, although not statistically different (*p* = 0.253) due to a small number of cases. However, this result suggests that nodules with more than one type of echogenic foci should be considered suspicious and biopsied.

There are several limitations in our study. First, the appearance of echogenic foci is subjective nature and dependent on factors such as ultrasound machine settings. It is a somewhat dynamic phenomenon that is often more evident on real-time scans. Second, for benign lesions, surgical confirmations were not done and were based on cytopathologic results; this may cause false-negative or false-positive results. Finally, we did not assess the role of isolated echogenic foci or macrocalcification alone as a predictor of malignancy. A previous study demonstrated that thyroid nodules with isolated macrocalcification had a low to intermediate malignancy risk, with a range of 11.4 to 16.1% [23].

## Conclusion

Overall, the presence of echogenic foci in thyroid nodules is associated with higher rate of malignancy compared to nodules without echogenic foci. Our study showed the usefulness of various TIRADS and its applicability in nodules with six types of echogenic foci. In conclusion, combination with TIRADS could offer better stratification of the malignancy risk of thyroid nodules than individual US feature of echogenic foci and may provide more evidence-based recommendations to patients.

## Additional file

Additional file 1: **Table S1.** US characteristics of benign and malignancy thyroid nodules with echogenic foci (*n* = 390). **Table S2.** Histopathologic Results of 417 malignancies. (DOCX 16 kb)

## Abbreviations

ACR: American College of Radiology
CNB: Core-needle biopsy
FNA: Fine-needle aspiration
KSThR: Korean Society of Thyroid Radiology
NPV: Negative predictive value
PPV: Positive predictive value
TIRADS: Thyroid Imaging Reporting and Data System
US: Ultrasonography
