# Supplementary material for: Echogenic foci in thyroid nodules: diagnostic performance with combination of TIRADS and echogenic foci
Source: BMC Med Imaging. 2019 Apr 4;19:28. doi: 10.1186/s12880-019-0328-2 (PMC6449957; doi:10.1186/s12880-019-0328-2)
Supplement: Supplementary file 1 — Table S1. US characteristics of benign and malignancy thyroid nodules with echogenic foci (n = 390). Table S2. Histopathologic Results of 417 malignancies. (DOCX 16 kb) [file 12880_2019_328_MOESM1_ESM.docx]

**Supplementary Table S1** US characteristics of benign and malignancy thyroid nodules with echogenic foci (n=390)

| Characteristics | | Benign (n = 136) | Malignant (n = 254) | p value |
| --- | --- | --- | --- | --- |
| Internal content | | | | |
|  | Solid | 114(83.8) | 238 (93.7) |  |
|  | Predominantly solid | 22 (16.2) | 16 (6.3) | 0.984 |
| Shape | | | | |
|  | Ovoid to round | 79 (58.1) | 61 (24.0) | 0.038 |
|  | Irregular | 41(30.1) | 69 (27.2) | 0.854 |
|  | Taller than wide | 16 (11.8) | 124 (48.8) | 0.026 |
| Margin | | | | |
|  | Well-defined smooth | 40 (29.4) | 14 (5.5) | <0.001 |
|  | Ill-defined | 95 (69.9) | 145 (57.1) | 0.002 |
|  | Spiculated/microlobulated | 1 (0.7) | 95 (37.4) | <0.001 |
|  | | | | |
| Echogenicity | Isoechogenicity | 50 (36.8) | 13 (5.1) | <0.001 |
|  | Hypoechogenicity | 76 (55.9) | 175 (68.9) | <0.001 |
|  | Marked hypoechogenicity | 9 (6.6) | 64 (25.2) | <0.001 |
|  | Hyperechogenicity | 1 (0.7) | 2 (0.8) | 0.330 |
|  | | | | |

Note. Data in parentheses are percentages

**Supplementary Table S2** Histopathologic Results of 417 malignancies

| Histopathologic Result | Nodules without echogenic foci (n=163) | Nodules with echogenic foci (n=254) |
| --- | --- | --- |
| Papillary carcinoma |  |  |
| Conventional | 124 (76.1) | 205 (80.7) |
| Follicular variant | 22 (13.5) | 32 (12.6) |
| Follicular carcinoma | 3 (1.8) | 6 (2.4) |
| Other malignancy | 14 (8.6) | 11 (4.3) |

Note. Data in parentheses are percentages
